# Pleural Infection: Contemporary Microbiology Completing the Picture

**DOI:** 10.1093/cid/ciae103

**Published:** 2024-03-06

**Authors:** Ruben Dyrhovden, Tomas Mikal Eagan, Øyvind Kommedal

**Affiliations:** Department of Microbiology, Haukeland University Hospital, Bergen, Norway; Department of Clinical Science, University of Bergen, Bergen, Norway; Department of Thoracic Medicine, Haukeland University Hospital, Bergen, Norway; Department of Microbiology, Haukeland University Hospital, Bergen, Norway

To the  Editor—We appreciate the opportunity to respond to the letter by Musher et al. [1] in which they critically assess our investigation on the microbiology of community-acquired oral-type pleural infections (OPIs). Unfortunately, they seem to ignore that we are addressing a very specific group of pleural infections and substantiate their criticisms by referring to publications inadequate to the task. The 1972 study by Bartlett and Finegold described anaerobic empyema exclusively [2]. The 1974 study by Bartlett et al. excluded patients that had received antibiotics [3]. Neither study distinguished between community-acquired and hospital-acquired infections, and their findings are not directly comparable to ours.

The referred studies were performed prior to the advent of next-generation sequencing (NGS). NGS has exposed the inadequacy of culture for describing complex bacterial infections, in particular those involving anaerobic bacteria or if patients have received antibiotics before sample collection. Pleural infections are often complex, frequently contain anaerobes and many patients have received antibiotics before sample collection. For pleural infections, studies using NGS report that culture recovered 10%–16% of species present and that 50%–78% of samples positive by NGS remained culture negative [4–6].

We claimed our study to be the first specifically designed for investigating the epidemiology, microbiology, clinical characteristics, and risk factors associated with OPIs [4]. We refer to several papers that have found *Streptococcus intermedius* and *Fusobacterium nucleatum* to be common in pleural infections. However, to suggest they are key pathogens necessary for establishing the infection, complete descriptions of all samples in a well-defined population is imperative. This can only be achieved using molecular methods.

We provide strong arguments to support hematogenous seeding from an oral focus as the underlying etiology in OPIs [4]. We show that the most common bacteria in OPI are also the most common in dental infections, that they can be cultured from blood, and are able to spread hematogenously to the brain to create abscesses with a microbiology indistinguishable from that of the pleural infections. Additional evidence includes multiple reports of patients having concomitant brain-abscess and lung abscess or empyema with *F. nucleatum* or *S. intermedius* [7, 8]. Hematogenous seeding also explains the thoroughly documented existence of pleural empyema with no evidence of associated pneumonia [5, 9]. Importantly, several patients in our cohort had isolated periapical infections, requiring examination by an oral surgeon to be revealed. Even edentulous patients can still have residual infected roots, especially in some populations at risk for empyema [10].

Bacteria associated with OPI are not normally involved in pneumonia. In recent papers on the etiology of community acquired pneumoniae by Musher and colleagues, there is no mention of the most common bacteria in OPI [11, 12]. Aspiration pneumonia is today considered typically polymicrobial, dominated by aerobic gram-negative bacilli, whereas the role of anaerobes is doubted [13, 14]. As discussed in our paper, populations at risk for aspiration are overlapping with populations at risk for poor dental health and for having a predisposing focus in the lung. Older animal studies are sometimes referred in support of aspiration [2]. A series of animal studies was conducted at the time, summarized by Longacre and Herrmann [15]: “In short, both clinical and laboratory experience has demonstrated the lung to be very susceptible to the formation of abscess from septic embolism and to be very resistant to bronchogenic inoculation.”

While preparing this response, we were excited to discover that other studies also assume the existence of a predisposing focus in the lung, report a correlation with poor oral health, and consider lung abscess, necrotic pneumonia, and anaerobic empyema different manifestations of the same disease [15, 16].
